# *EGFR* Blockade Reverses Cisplatin Resistance in Human Epithelial Ovarian Cancer Cells

**DOI:** 10.29252/ibj.24.6.365

**Published:** 2020-03-30

**Authors:** Arash Poursheikhani, Hassan Yousefi, Javad Tavakoli-bazzaz, Seyed H. Ghaffari

**Affiliations:** 1 Medical Genetics Research Centre, Faculty of Medicine, Mashhad University of Medical Sciences, Mashhad, Iran;; 2 Hematology/Oncology and Stem Cell Transplantation Research Center, Shariati Hospital, School of Medicine, Tehran University of Medical Sciences, Tehran, Iran;; 3 Louisiana State University, School of Medicine, New Orleans, USA;; 4 Department of Medical Genetics, School of Medicine, Tehran University of Medical Sciences, Tehran, Iran

**Keywords:** Cisplatin, Epidermal growth factor receptor, Ovarian cancer

## Abstract

**Background:**

EOC is one of the most lethal gynecological malignancy worldwide. Although the majority of EOC patients achieve clinical remission after induction therapy, over 80% relapse and succumb to the chemoresistant disease. Previous investigations have demonstrated the association of *EGFR* with resistance to cytotoxic chemotherapies, hormone therapy, and radiotherapy in the cancers. These studies have highlighted the role of *EGFR* as an attractive therapeutic target in cisplatin-resistant EOC cells.

**Methods:**

The human ovarian cell lines (SKOV3 and OVCAR3) were cultured according to ATCC recommendations. The MTT assay was used to determine the chemosensitivity of the cell lines in exposure to cisplatin and erlotinib. The qRT-PCR was applied to analyze the mRNA expression of the desired genes.

**Results:**

Erlotinib in combination with cisplatin reduced the cell proliferation in the chemoresistant EOC cells in comparison to monotherapy of the drugs (*p *< 0.05). Moreover, erlotinib/cisplatin combination synergistically decreased the expression of anti-apoptotic and also increased pro-apoptotic genes expression (*p *< 0.05). Cisplatin alone could increase the expression of MDR genes. The data suggested that EGFR and cisplatin drive chemoresistance in the EOC cells through MEKK signal transduction as well as through EGFR/MEKK pathways in the cells, respectively.

**Conclusion:**

Our findings propose that *EGFR* is an attractive therapeutic target in chemoresistant EOC to be exploited in translational oncology, and erlotinib/cisplatin combination treatment is a potential anti-cancer approach to overcome chemoresistance and inhibit the proliferation of the EOC cells.

## INTRODUCTION

EPithelial ovarian cancer is one the most common cancer among gynecological malignancy and the fifth leading cause of cancer-related death among women globally^[^^1^^]^. It was estimated that nearly 14000 patients died from EOC, and 22000 new cases were diagnosed with EOC in the United States in 2018^[^^2^^]^. Advanced-stage diagnosis, peritoneal dissemination, and the establishment of therapy resistance are the major obstacles for the successful treatment of EOC and contribute to marginal overall survival rate^[^^3^^]^. 

Conventional treatment strategies for EOC includes radical debulking surgery, following by platinum-taxane-based regimens and adjuvant chemotherapies^[3]^. Despite advances in surgical debulking and chemotherapy regimens, over 80% of patients with late-stage disease relapse within a few months due to resistance to both chemotherapeutic agents and targeted therapies^[^^4^^]^. 

A large body of evidence has suggested that the ErbB family of receptor tyrosine kinases has a significant contribution to the initiation and progression of a variety of human malignancies^[^^5^^]^. The EGFR, a member of ErbB family of receptor tyrosine kinases, activates multiple downstream signaling pathways, including *Ras*/*Raf*/*MAPK* and *PI3K/Akt,* thereby promoting the proliferation, invasion, and metastasis of tumor cells^[^^6^^]^. Previous investigations have demonstrated that *EGFR* overexpression has been associated with resistance to cytotoxic chemotherapies, hormone therapy, and radiotherapy^[^^7^^,^^8^^]^. *EGFR* overexpression has been observed in 30–98% of EOC in all histologic subtypes^[^^9^^]^. Enhanced expression of *EGFR* and its ligands are correlated with advanced-stage disease, poor response to chemotherapies, dismal clinical outcome, and decreased recurrence-free survival^[^^10^^]^. Preclinical studies with cetuximab (an anti-*EGFR* mAb) as well as gefitinib and erlotinib (*EGFR* small molecule inhibitors) have displayed that *EGFR*-targeted therapies enhance the anti-tumor activity of the chemotherapeutic agents and radiotherapy in colorectal, pancreatic, non-small cell lung, and breast cancer cells^[^^11^^-^^14^^]^. These studies support the hypothesis that the inhibition of the *EGFR* pathway in combination with the chemotherapies might strengthen the antitumor activity of mentioned agents, leading to the increased apoptotic cell death. 

Erlotinib is a reversible and highly specific EGFR tyrosine kinase inhibitor that is orally administrated in a variety of cancers^[^^15^^]^. Several randomized clinical trials have evaluated the efficacy and benefit of erlotinib in cancer, particularly in non-small cell lung cancer^[^^16^^]^. Cisplatin is also one of the most commonly used platinum-based chemotherapy agent administrated as the first-line standard treatment for EOC and in a broad range of cancers^[^^17^^]^. Cisplatin has been indicated to bind to the cellular components such as DNA and protein and inhibits molecular processes such as DNA replication and protein translation via forming DNA-cross link in the cells^[^^18^^]^. In the present study, we demonstrated the *in vitro* activity of erlotinib in the EOC cell lines and showed that *EGFR* blockade restores cisplatin sensitivity in these cells.

## MATERIALS AND METHODS

**Drugs**

Erlotinib (anti-*EGFR*) and buparlisib (anti-*PI3K*) were purchased from Chemietek (Indianapolis, IN, USA) and Bay 11-7082 (anti- *NFκB*) and trametinib (anti-*MEKK1/MEKK2*) from Sigma (St. Louis, MO, USA). Cisplatin (DNA-damaging agent) was also acquired from the Pharmacy of Shariati Hospital (Tehran, Iran). 

**Human ovarian carcinoma cell lines**

Human ovarian carcinoma cell lines, SKOV3 and OVCAR3, were obtained from the National Cell Bank of Iran, Pasteur Institute of Iran, Tehran. The cells were cultured, according to ATCC recommendations, in RPMI-1640 medium supplemented with 10% fetal bovine serum and 1% penicillin-streptomycin antibiotics (both from Gibco, Life Technologies, USA) in a humidified incubator in 5% CO_2_ at 37 °C.

**Anti-proliferative assays**

The EOC cells were cultured in 96-well plates (2 × 10^3^ cells/well). After incubation at 37 °C for 24 h, the cultures were treated with desired concentrations of the chemotherapy agents. Following 48 h of treatment, the proportion of viable cells was determined by MTT assay. After 48 and 72 hours, 20 µl of MTT solution (5 mg/ml; Sigma) was added to each well and incubated with 5% CO_2 _at 37 °C for 4 hours. The supernatant was removed, and 100 µl of DMSO was added to each well as a solvent. Cell viability percentage was assessed by spectrophotometry at 570 nm using Absorbance Microplate Reader (BioTek ELx800, USA). The cytotoxicity was reported as IC_50_ values calculated from full dose-response curves. Synergism was determined by drawing the normalized isobologram according to Chou-Talalay method (reviewed in^[^^19^^]^) using the Calcusyn software (Biosoft, Cambridge, UK). Untreated EOC cells were considered as the positive control, and blank wells were regarded as the negative control. 

**RNA extraction and cDNA synthesis**

Trizol (Life Technologies, USA) was used to harvest the total RNA from the cultured cells. The quantity and quality of the extracted RNA were investigated by the 2% gel electrophoresis method. Then RNA concentration was measured by a Nanodrop (Thermo Scientific, USA). cDNA was prepared by the PrimeScript RT cDNA synthesis kit (Takara Bio, USA) according to the manual instruction. The total volume for this reaction was 20 μl that included 2 µg of total RNA, 4 μl of 5× buffer, 1 μl of dNTP, 1 μl of RNase Inhibitor, 2 μl of random hexamers, 1 μl of Moloney Murine Leukemia Virus  *reverse transcriptase*, and DEPC water.

**Analysis of gene expression by qRT-PCR**

Changes in mRNA levels of the desired genes were measured by qRT-PCR on a LightCycler® 96 System (Roche Life Science, Germany) using SYBR green-based kit, RealQ Plus Master Mix Green (Ampliqon, Copenhagen, Denmark). The total volume was 20 μL, including 10 μL of SYBR Green, 1 μL of primer, 2 μL of cDNA, and DEPC water. Thermal cycling conditions were comprised of an activation step at 95 °C for 15 min, followed by 40 cycles, including a denaturation step at 95 °C for 10 seconds and at 60 °C for 1 min for annealing and extension, respectively. The primer sets are listed in Table 1. The target gene expression levels were normalized to *HPRT1* levels. For calculation of relative expression, 2^–ΔΔCT^ formula was used. *HGPRT1* gene expression was considered as the positive control, and DEPC water was considered as the negative control. 

**Statistical analysis**

The data were graphed and analyzed using GraphPad Prism (version 6.01). Unpaired two-tailed Student’s *t*-test and One-way ANOVA were used for comparing the means between two and more than two independent groups, respectively. Heatmap was drawn via R software (3.5.1). All data were presented as mean ± SD, and all the experiments were conducted in triplicate for three independent times. A *p *value <0.05 was considered as statistically significant threshold. 

**Table 1:** Nucleotide sequences of the primers used for qRT-PCR

**Gene**	**Accession number**	**Forward primer**	**Reverse primer**	**Amplicon** **size (bp)**
*HPRT1*	NM_000194	GGACAGTACGGGAGATCACAG	GCACTAATTTCCTTCAGGGATCG	111
*FOXO1*	NM_002015	TGATAACTGGAGTACATTTCGCC	CGGTCATAATGGGTGAGAGTCT	80
*FOXO3*	NM_001455	ACGGCTGACTGATATGGCAG	CGTGATGTTATCCAGCAGGTC	85
*FOXO4*	NM_005938	CACGTATGGATCCGGGGAAT	CCCCTCCGTGTGTACCTTTTC	191
*P21*	NM_000389	CCTGTCACTGTCTTGTACCCT	GCGTTTGGAGTGGTAGAAATCT	130
*P27*	NM_004064	AACGTGCGAGTGTCTAACGG	CCCTCTAGGGGTTTGTGATTCT	209
*BAX*	NM_001291428	CGAGAGGTCTTTTTCCGAGTG	GTGGGCGTCCCAAAGTAGG	242
*BCL2*	NM_000633	CGGTGGGGTCATGTGTGTG	CGGTTCAGGTACTCAGTCATCC	90
*ABCG2*	NM_004827	TGAGCCTACAACTGGCTTAGA	CCCTGCTTAGACATCCTTTTCAG	75
*ABCB1*	NM_000927	TTGCTGCTTACATTCAGGTTTCA	AGCCTATCTCCTGTCGCATTA	105
*ABCC*	NM_004996	CTCTATCTCTCCCGACATGACC	AGCAGACGATCCACAGCAAAA	94
*cIAP1*	NM_001166	AGCACGATCTTGTCAGATTGG	GGCGGGGAAAGTTGAATATGTA	102
*XIAP*	NM_001167	ATAGTGCCACGCAGTCTACAA	AGATGGCCTGTCTAAGGCAAA	101
*MCL1*	NM_021960	TGCTTCGGAAACTGGACATCA	TAGCCACAAAGGCACCAAAAG	135
*CCND1*	NM_053056	GCTGCGAAGTGGAAACCATC	CCTCCTTCTGCACACATTTGAA	135
*MYC*	NM_002467	GTCAAGAGGCGAACACACAAC	TTGGACGGACAGGATGTATGC	162
*Survivin*	NM_001168	CCAGATGACGACCCCATAGAG	TTGTTGGTTTCCTTTGCAATTTT	152
*EGF*	NM_001963	TGTCCACGCAATGTGTCTGAA	CATTATCGGGTGAGGAACAACC	133
*HB-EGF*	NM_001945	ATCGTGGGGCTTCTCATGTTT	TTAGTCATGCCCAACTTCACTTT	86
*AREG*	NM_001657	GAGCCGACTATGACTACTCAGA	TCACTTTCCGTCTTGTTTTGGG	121
*EREG*	NM_001432	GTGATTCCATCATGTATCCCAGG	GCCATTCATGTCAGAGCTACACT	120
*BTC*	NM_001729	CCTGGGTCTAGTGATCCTTCA	CTTTCCGCTTTGATTGTGTGG	131
*HER3*	NM_001982	GGTGATGGGGAACCTTGAGAT	CTGTCACTTCTCGAATCCACTG	80
*HER2*	NM_001005862	CAGTGCAGCACAGAGACTCA	CCGGTGCACACTCACTTTTG	103
*EGFR*	NM_001346897	AGACAGCTTCTTGCAGCGAT	TTCCAGACAAGCCACTCACC	105
*HRG1A*	NM_013964	AAACCAAGAAAAGGCGGAGGAGCT	GAGGGCGATGCAGATGCCGG	70
*HRG1B*	NM_013956	GCCAGCTTCTACAAGCATCTTGGGA	GGAGGGCGATGCAGATGCCG	97

## RESULTS

**Chemoresistance of the EOC cell lines**

**Fig. 1:**
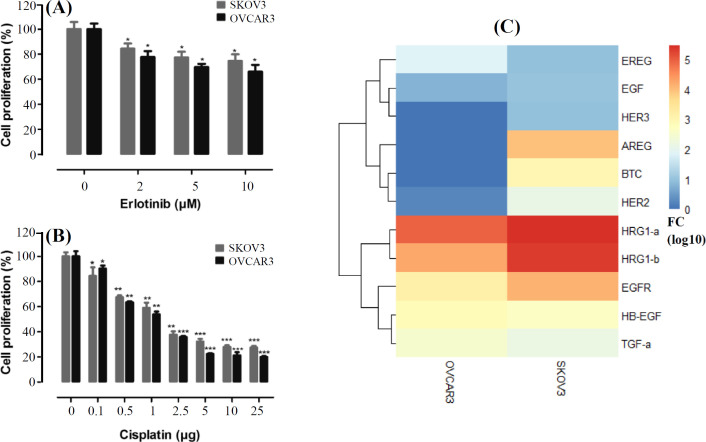
Erlotinib and cisplatin resistance profiles and the relative expression of EGF ligands and receptors. (A and B) SKOV3 and OVCAR3 cells exposed to erlotinib (2, 5, and 10 μM) and cisplatin (0.1, 0.5, 1, 2.5, 5, 10, and 25 µg). The data are presented as mean ± SD; (C) ErbB family expression profile in the cells. ^*^p < 0.05, ^**^p < 0.01, and ^***^p < 0.001

Since cisplatin is considered to be the first line conventional therapy in EOC, we tried to test the effects of cisplatin in combination with erlotinib on the cell viability of EOC cell lines. Chemoresponsiveness of the EOC cell lines, SKOV3 and OVCAR3, to cisplatin and erlotinib were measured by MTT assay and are presented in Figure 1A and 1B. The IC_50_ of cisplatin for SKOV3 and OVCAR3 was as 1.84 μg and 1.44 μg, respectively. These amounts were as 58.57 μM and 53.49 μM for erlotinib. 

**Expression of** **ErbB family** **in the EOC cells**

To explore the expression of ErbB ligands and receptors, the relative expression of *EREG, EGF*,* HER3,* *AREG*, *BTC*, *HER2, HRG-1A, HRG-1B, EGFR, HB-EGF*, and *TGF-α *were investigated in SKOV3 and OVCAR3 cell lines by qRT-PCR. This screening experiment demonstrated that the expression of the *ErbB *family members is relatively expressed in those cells (Fig. 1C).

**Effect of erlotinib on the EOC cells**

**Fig. 2:**
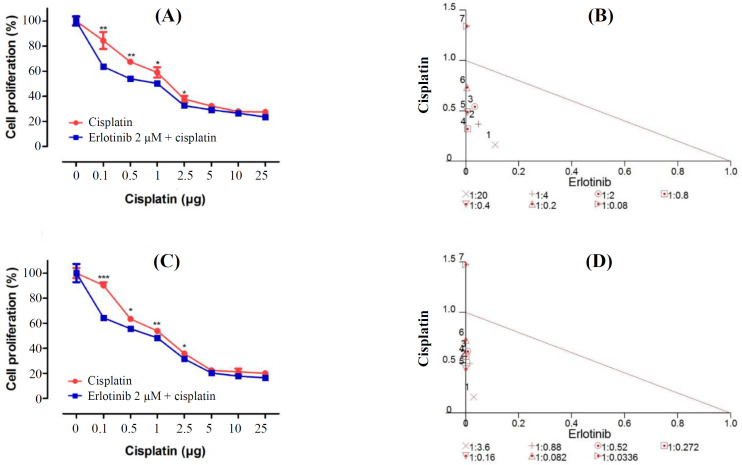
Synergistic effects of erlotinib and cisplatin on SKOV3 and OVCAR3 cell lines. (A) SKOV3 and (C) OVCAR3. (B and D) Normalized isobologram analysis represents the synergic effect of erlotinib (2 µM) and cisplatin (0.1, 0.5, 1, 2.5, 5, 10, and 25 µg) when using combination treatment in SKOV3 and OVCAR3 cell lines. The combination index was calculated with Calcusyn software. Points above, below, and over the isobologram effect line reflect antagonism, synergy, and additive effect, respectively. The numbers under the isobolograms indicate the doses of erlotinib and cisplatin in combination. Statistically significant values of ^*^p < 0.05, ^**^p < 0.01, and ^***^p < 0.001 were determined compared with the control

The effects of erlotinib in combination with cisplatin on the proliferative response of SKOV3 and OVCAR3 cells were evaluated. This combinatorial approach had synergistic effects on the growth inhibition. Cisplatin significantly reduced cell proliferation in combination with 2 μM of erlotinib, dose-dependently (*p *< 0.05). Moreover, 0.1 μg of cisplatin showed noticeable combinatorial effect with 2 μM of erlotinib in the EOC cells (*p *< 0.001). According to the normalized isobologram, 0.1 μg of cisplatin had the most powerful synergism with 2 μM of erlotinib in EOC cells. It has been suggested that erlotinib-mediated inhibition of *EGFR* increases the cisplatin responsiveness in SKOV3 and OVCAR3 cells (Fig. 2). For further investigation, 0.1 μg of cisplatin and 2 μM of erlotinib were chosen. 

**Effects of erlotinib/cisplatin combinatorial therapy**

**Fig. 3:**
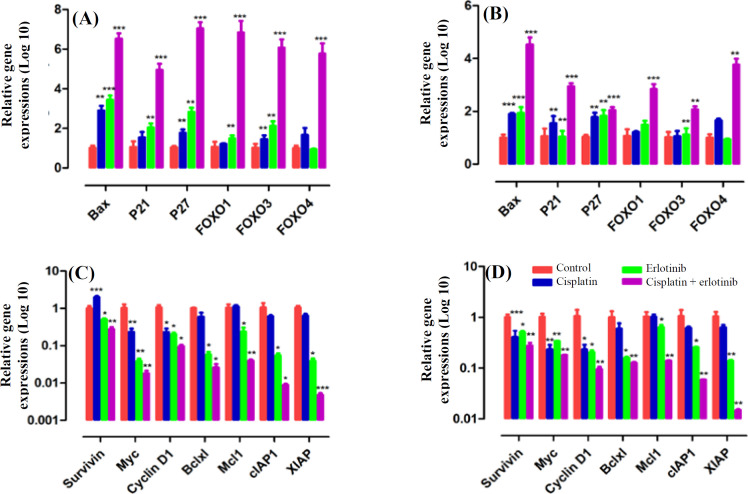
Erlotinib and cisplatin combination treatment inducing apoptosis and reducing anti-apoptotic gene expression. The EOC cells were treated with cisplatin (0.1 μg) and erlotinib (2 μM) for 48 h, then total RNA was harvested for qRT-PCR analysis. Gene expression levels were normalized to *HPRT1*. Erlotinib in combination with cisplatin dramatically diminished the gene expression of pro-apoptotic genes in SKOV3 (A) and OVCAR3 (B) cells. Anti-apoptotic genes expression levels were meaningfully depleted in erlotinib/cisplatin treatment in SKOV3 (C) and OVCAR3 (D) cell lines. ^*^p < 0.05, ^**^p < 0.01, and ^***^p < 0.001

To demonstrate the anti-tumor effects of cisplatin in combination with erlotinib on the EOC cells, we investigated the effects of erlotinib, cisplatin, and combinatorial treatment on the expression of pro-apoptotic and anti-apoptotic genes. Accordingly, SKOV3 and OVCAR3 cells were exposed to cisplatin (0.1 μg) and erlotinib (2 μM) for 48 h. Erlotinib/ cisplatin combination treatment remarkably increased mRNA levels of pro-apoptotic genes such as *BAX*,* p21*,* p27*,* FOXO1*,* FOXO3*, and *FOXO4*, nearly five times in comparison to monotherapy of cisplatin or erlotinib (*p *< 0.001)*. *Furthermore, we observed a significant reduction in the mRNA levels of anti-apoptotic genes, including *survivin*,* MYC*,* Cyclin D1*,* BCL-xl*, *MCL1*,* cIAP1*, and* XIAP*, approximately ten times in comparison with the controls and the chemotherapy alone (*p *< 0.01; Fig. 3).

**Effect of chemoresistance through** ***EGFR/MEKK*** **pathway**

**Fig. 4:**
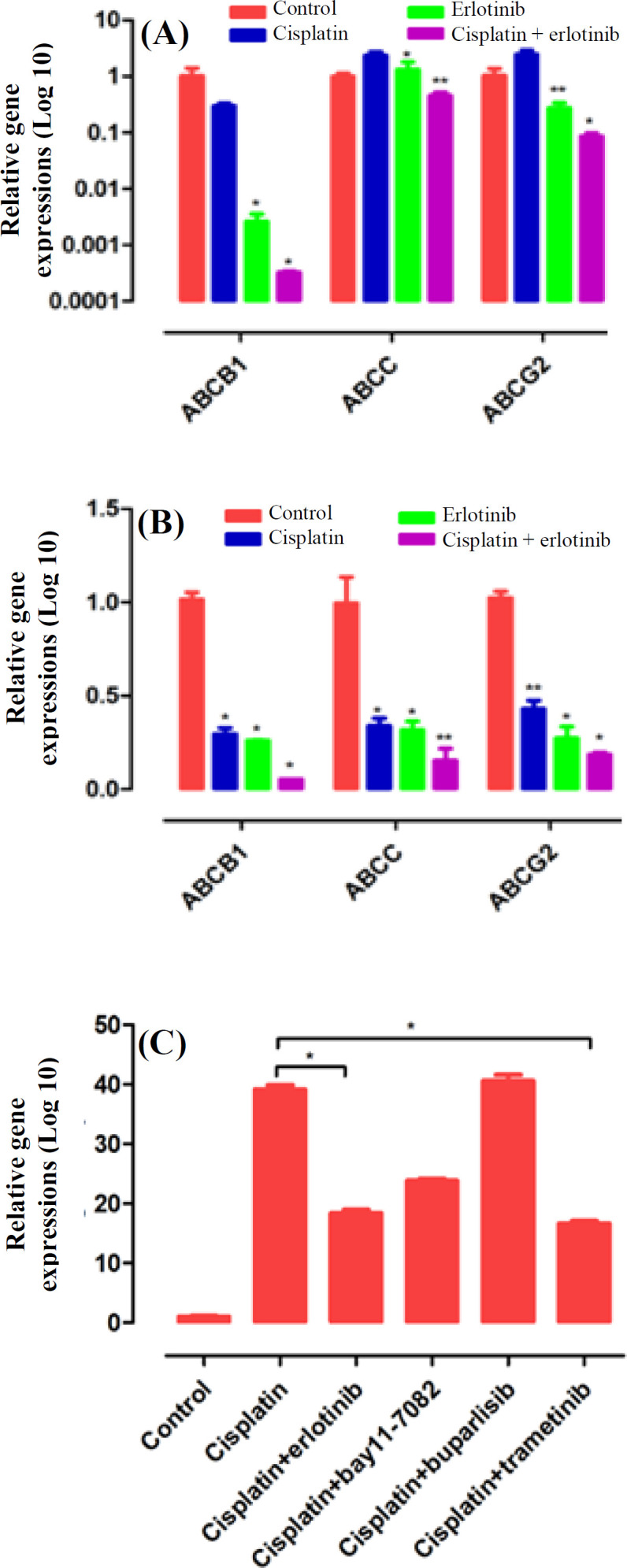
Cisplatin enhancement by chemoresistance through *EGFR/MEKK *pathway. The SKOV3 (A) and OVCAR3 (B)  cell lines were treated with cisplatin (0.1 μg) and erlotinib (2 μM) for 48 h, then total RNA was harvested for qRT- PCR analysis. Erlotinib/cisplatin combination significantly reduced MDR genes in both cell lines with respect to the control. (C) SKOV3 cells were exposed to cisplatin (0.1 μg) as an igniter of chemoresistance, erlotinib (2 μM) as an anti-*EGFR*, bay11-7082 (5 μM) as an anti-*NFκB*, buparlisib (1 μM) as an anti-*PI3K*, and trametinib (5 μM) as an anti*-MEKK1/MEKK2* for signaling dissection. After 48 h, RNA was harvested for qRT-PCR. *HB-EGF* (*EGFR* ligand) was evaluated in the treated cells

Cisplatin underlying chemoresistance mechanisms in the EOC cell lines was investigated by treating SKOV3 and OVCAR3 cells with cisplatin (0.1 μg) and erlotinib (2 μM) for 48 h. Erlotinib/cisplatin combination treatment significantly reduced mRNA levels of MDR genes (*ABCB1*,* ABCC*, and *ABCG2*) compared to the control and the single-agent treatments (*p *< 0.05). Cisplatin alone marginally increased the MDR genes expression levels in the SKOV3 cells (*p *< 0.07, Fig. 4A and 4B). Furthermore, we explored how cisplatin enhanced chemoresistance in cancerous cell lines. The mRNA levels were analyzed by qRT-PCR. The data showed that cisplatin alone significantly increased *HB-EGF* (EGFR ligand) in the SKOV3 cells. *HB-EGF* expression just reduced in the cisplatin/erlotinib and cisplatin/trametinib combinatorial approaches significantly (*p *< 0.05), which showed that EGFR was driving chemoresistance in the EOC cells through *MEKK1/MEKK2* signal transduction. The reduction of *HB-EGF* in the combinatorial approaches was so similar (two times) and significant. The data suggest that cisplatin drives chemoresistance through *EGFR/MEKK *pathways in the target cells (Fig. 4C). 

## DISCUSSION

Despite advances in surgical debulking and chemotherapy regimens, EOC has exhibited marginal improvement in survival. Although most patients achieve a clinical remission after the induction therapy, resistance to chemotherapy will occur subsequently. Moreover, relapsed tumors have a poor response to other cytotoxic agents, as well. Hence, in order to improve the outcome of the EOC patients, it is of paramount importance to devise novel and more efficient therapies aimed at blocking pivotal signaling pathways responsible for therapy resistance^[^^20^^]^. Alternation in cellular signaling pathways after chemotherapy treatment may lead to the initiation of drug resistance^[^^21^^]^. 

EGFR pathway is a key regulator of chemoresponsiveness in human malignancies. It has been suggested that cisplatin can interact with the EGFR signaling pathway and could either promote or inhibit apoptosis^[^^22^^]^. A previous study has revealed that cisplatin-resistant human ovarian cancer cell lines have an enhanced rate of motility and invasion *in vitro*, which is associated with hyperactivation of EGFR^[^^23^^]^. To better clarify the events happening in the EGFR signaling pathway after cisplatin treatment and their roles in promoting chemoresistance in the EOC cells, we applied *in vitro* models of the EOC cells and found that cisplatin in combination with erlotinib synergistically reduced the cell growth ability of those cells. Expression data showed that erlotinib/cisplatin combination treatment dramatically decreased the mRNA levels of anti-apoptotic genes compared to the control*. *Studies have highlighted the role of BCL2 family and cyclin proteins in the development of chemoresistance in many types of cancers. It has also been displayed that *BCL2* upregulation was associated with cisplatin-resistance in bladder, ovarian, lung, and breast cancer^[^^24^^,^^25^^]^. Downregulation of *BCL2* and *cyclin D1* enhanced cisplatin sensitivity in breast cancer cell lines^[^^26^^]^. A large body of evidence has demonstrated that the overexpressions of *MYC*, *XIAP*, and *surviving *are correlated with platinum and taxol resistance of the EOC cells^[^^27^^,^^28^^]^. In addition, our data showed that erlotinib in combination with cisplatin significantly up-regulated the expression of pro-apoptotic genes, including *BAX*,* p21*,* p27*, *FOXO1*,* FOXO3*, and *FOXO4*, compared to the control and the single-agent regiment. Vast evidence has revealed that the reduced expression of *p27* and *p21* is associated with chemotherapy resistance and poor prognosis in the patients^[^^29^^,^^30^^]^. *BAX* gene expression has been shown to associate with an increased progression-free and overall survival of the EOC patients^[^^31^^]^. A previous study has considered the forkhead transcription factor FOXO1 as a transcriptional factor, which can develop drug resistance in ovarian cancer^[^^32^^]^. EGFR drives proliferation, MDR genes expression, which efflux cisplatin out of the cells, cell survival, and apoptosis inhibition, ultimately resulting in chemoresistance in the cells. We showed that erlotinib induced apoptosis, reduced cell proliferation, and downregulated MDRs expression via blocking of EGFR. 

Further to erlotinib-mediated potential anti-tumor activity, down-regulation of MDRs could lead to the accumulation of cisplatin in the cells and enhance cisplatin chemotherapeutic effects. One study showed that trametinib (*MEKK* inhibitor) remarkably reduced the cell growth and proliferation in combination with vincristine and doxorubicin through blocking the drug-efflux activity of *ABCB1* transporter^[^^33^^]^. Cisplatin-resistant cells intrinsically up-regulate ATP-binding cassette transports (*ABC*) to efflux chemotherapy agents, consequently reducing the efficacy of the chemotherapies^[^^34^^]^. In addition, erlotinib can reduce drug efflux through the downregulation of ABC pump^[^^35^^]^. Phase 2 trial conducted by Nogueira‐ Rodrigues *et al.*^[^^36^^] ^displayed that treatment with erlotinib in combination with cisplatin plus radiotherapy exerted significant effects on cervical cancer patients and remarkably improved overall and progression-free survival. Some *in vitro* studies considered *EGFR* family and related signaling transduction as critical players in chemoresistance via the induction of MDR pumps^[^^37^^,^^38^^]^. Also, *EGFR* inhibitor PD153035 can synergistically increase the anti-tumor effect of chemotherapy modalities via down-regulating *ABCG2* in non-small cell lung cancer^[^^37^^]^. In fact, based on evidence, cisplatin treatment activates the *EGFR *and its downstream signaling pathway *MEKK1/MEKK2*, which has previously been shown to be implicated in chemoresistance in EOC^[^^38^^]^. 

Taken together, our results suggest that *EGFR *plays a key role in the development of chemoresistance in SKOV3 and OVCAR3 cells via the induction of MDR genes through the *EGFR/MEKK1*,* MEKK2* pathway. Oure studies highlight the role of *EGFR* as an attractive therapeutic target and suggest that *EGFR* blocking-therapies seem to be promising strategies against chemoresistant EOC. 

## CONFLICT OF INTEREST.

None declared.
